# Study on potential applications and toxicity analysis of green synthesized nanoparticles

**DOI:** 10.3906/kim-2106-59

**Published:** 2021-09-27

**Authors:** Rajni GARG, Priya RANI, Rishav GARG, Nnabuk Okon EDDY

**Affiliations:** 1 Department of Chemistry, University School of Sciences, Rayat Bahra University, Mohali, India; 2 Department of Civil Engineering, Galgotias College of Engineering and Technology, Greater Noida, India; 3 Department of Pure and Industrial Chemistry, University of Nigeria, Enugu State, Nigeria

**Keywords:** Nanomaterials, nanoparticles, green synthesis, antimicrobial action, toxicity analysis

## Abstract

Nanomaterials have garnered the significant interest of scientists owing to their technological as well as medical applications. In particular, metal and metal oxide nanoparticles have gained prominence because of their enhanced performance as compared to their bulk counterparts. Metal-supported nanomaterials are anticipated to make major contributions to solving today’s most challenging issues, like energy harvesting and environmental remediation. The incorporation of nanoparticles into sensors has significantly enhanced their precision and selectivity. With the advent of green chemistry, green synthetic techniques have been prioritized for the synthesis of single and multicomponent nanomaterials. In the current review, we have addressed the multidimensional applications of nanoparticles in various sectors, including surface coatings, biosensing, environmental remediation, energy devices, construction, and nano probing, etc. This study focuses on the categorization of nanomaterials according to their source, dimensions, and composition, along with the exploration of synthetic modes. The eco-friendly and cost-effective greener route for the synthesis of nanoparticles has been explored in detail. Further, the antibacterial and cytotoxic potential has been addressed, and toxicity analysis has been conducted. The study signifies the augmented potential of green synthesized nanoparticles that can prove as economically viable and eco-friendly alternatives to conventional materials.

## 1. Introduction

Nanotechnology plays an imperious role in the expansion of sustainable technologies for humanity and the environment by producing a wide range of materials at the nanoscale level [1–3]. The nanostructured materials that are produced through various physical, chemical, and biological techniques have significant applications in energy conversion, information storage, drug delivery and improvement of the quality of bulk materials for further use [4–6]. Nanomaterials show improved physico-chemical, thermo-mechanical, and optical properties in contrast to the bulk materials owing to their fine size and greater surface area [7,8]. Nanoparticles fill the gap between bulk substances and molecular structures with fine particle size (1–100 nm) and high divergency resulting in cutting-edge offerings in the area of environmental remediation, catalysis, sensing, communications, cosmetics, pharmaceuticals, energy storage, and generation, etc. [9–11]. The applicability of nanoparticles in combating a diversity of coronavirus in the current COVID-19 pandemic crisis is worth mentioning [12].

Traditionally, many physical and chemical approaches are used to produce nanoparticles with associated drawbacks involving time, energy, and cost [13–15]. More recently, biological methods involving microorganisms, enzymes, and plant extracts have been employed with the advent of green chemistry and sustainable development [16–19]. The significant ease of control and manipulation of their morphological characteristics and functionalities with variation in synthetic technique adds versatility to the nanoparticles [20,21]. Especially, the biocompatibility, multiple receptor binding, and fluorescence characteristics of metal nanoparticles offer their use in biosensing, bio labeling, and therapeutics [22–25]. The antibacterial, antifungicidal, and antioxidant potential of nanoparticles has revolutionized the medical sector by providing a solution to the issues of antibiotic resistance, side-effects of drugs with target-specific drug delivery in addition to increasing the market value of daily consumer products, textiles, and sports [26–28]. The emerging applications of nanoparticles in the chemical industry have provided new directions in synthesis, catalysis, light-emitting diodes, paints, coatings, and water purification [29–32]. The proper incorporation and tailoring of nanoparticles in a suitable matrix results in the formation of engineered nanoparticles that provide additional benefits [33–35]. However, the toxicity analysis of these highly significant nanomaterials is still an issue of concern [36,37].

The present paper introduces the classification and synthetic techniques of nanomaterials with special emphasis on green synthesis being simpler, eco-friendly, and cost-effective due to using nontoxic and biodegradable reductants. The review focuses on highlighting the emerging applications of nanoparticles that are replacing conventional materials. The study also explores the antimicrobial action and toxicity analysis of nanoparticles along with the underlying causes.

## 2. Classification of nanomaterials

Nanomaterials are usually classified as per their provenance, dimensions, and composition [5]. Various types of nanomaterials are found to originate from natural activities and intentional or unintentional anthropogenic activities [4]. Various natural activities like volcanic eruptions, forest fires, tidal waves, storms, etc. become the source of various ultrafine nanomaterials. These are also produced as by-products of industrial and agricultural processes especially in mining, exploitation of quarry materials, thermal power stations, combustion reactions, operation of jet engines, etc. [38] Discovering nanoparticles called fullerenes in space is quite intriguing. Biogenic nanomaterials are also found as magnetite and ferritin in bacteria and brain cells of human beings [39].

The nanomaterials are categorized as 0D, 1D, and 2D depending upon the dimensions in the nanoscale (1–100 nm). In 0D nanomaterials, the movement of electron confinement is in all three directions with all dimensions in nanoscale [7]. Various means have been introduced to fabricate 0D nanomaterials such as quantum dots and nanoparticles due to their significant use in LEDs, solar cells, lasers, etc. [40–42].

**Figure 1:**
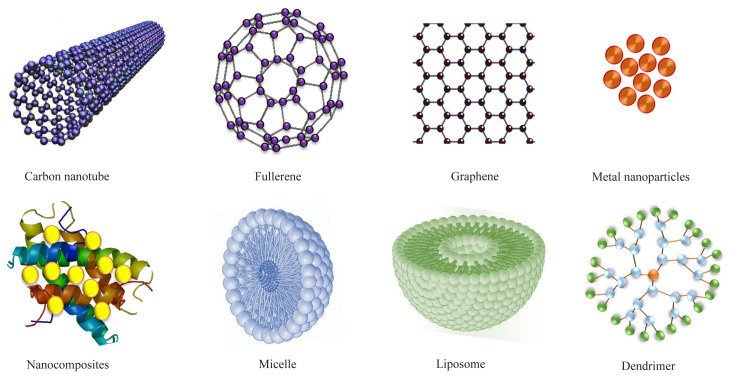
Nanomaterials of significant applications.

In the case of ID nanomaterials, the electron confinement is in two directions with one dimension not in nanoscale and include nanorods, nanowires, and nanofibers, etc. [30] 1D nanoparticles have attained meaningful attentiveness after the work on carbon nanotubes. 1D nanomaterials have a vast array of potential applications in nanoelectronics and nanodevices [7]. 2D nanomaterials exhibit the electron confinement in only one direction with two dimensions out of nanoscale and include nanoplates, nanocoatings, and thin films, etc. [43,44]. 2D nanoparticles with different geometries show various unique characteristics with shape-dependence [45]. Due to the electron movement in the X-Y plane, these are generally used to change the optical reflectance of the surfaces. Nanocoating of 2D nanomaterials is usually exhibited to improve the hardness and corrosion resistance by providing an insulating layer on the surface [29]. 3D nanomaterials with all dimensions out of nanoscale consist of nanocomposites and nanostructured materials such as aerogels, block copolymers, and alloys [26,46]. With free electron movement in all directions, these materials have a large specific area with high strength. 3D nanostructured are synthesized with controlled structure and morphological characteristics exhibiting an array of claims in the electronics, agri-food sector, energy storage devices, drug delivery, and catalysis, etc. [24,32,47–49]. Figure 1 illustrates some highly significant nanomaterials.

Carbon-based nanomaterials are composed of carbon units and include graphene, carbon nanofibers, carbon black, carbon nanotubes, and fullerenes [50]. Graphene comprises of one-atom thick carbon sheet used in electrodes, storage of electron and hydrogen, sensors and reinforcements, etc. [50]. Carbon nanotubes are elongated self-aligned graphene sheets rolled into a single or multiwalled cylindrical shape. The significant applications include scaffolds for tissue engineering, biosensing, and reinforcement material [51]. Carbon nanofiber is the irregular form of carbon nanotubes, while carbon black is the amorphous form of carbon obtained by incomplete combustion of hydrocarbons and are used to obtain composites [30,52]. Fullerenes are the cage-like structures of graphene sheets wrapped in the spherical or ellipsoidal form [4]. Fullerenes have very high strength, electrical conductivity, and electron affinity leading to their use in electronics, medical imaging, photovoltaics, catalysis, and environmental remediation. Functionalized hydrophilic fullerenes have significant potential in diagnostics and therapeutic applications [36].

Polymeric nanomaterials are organic-based materials in the shape of nanospheres or nanocapsules with improved electrical, thermal, mechanical, optical, and magnetic properties [40]. Polymeric nanoparticles can be operationalized with varied functionalities and, therefore, exhibit a wide range of applications [27]. Micelles and liposomes are the distinct type of copolymeric nanomaterials made up of hydrophilic and hydrophobic entities used in drug delivery, sensors, diagnostics, and phase-transfer catalysis [8,40,53]. Dendrimers are highly ordered hydrophilic polymeric and hyperbranched molecules that are extensively used in biomedical applications [54]. Lipid-based nanomaterials are spherical moieties made up of lipid core matrix stabilized by emulsifiers or surfactants and are used to encapsulate oligonucleotides. Lipid nanoparticles are effectively used in the biomedical field for drug delivery and cancer therapy [55].

Inorganic nanomaterials comprise semiconductors, pure metals, or their oxides. Metal nanoparticles are widely explored in light-emitting diodes, photovoltaics, and optoelectronics due to their advanced optical, electrical, and magnetic properties [10,39]. E.g., Silver and gold nanoparticles are used in optical, fuel cells, electronics, medical diagnostics, drug delivery, antibacterial applications, etc. [56–59]. Nanomaterials of metal oxides viz titanium oxide and zinc oxide are exploited as antibacterial agents, photocatalysts, and self-cleaning agents in cosmetics, filtration units, and solar cells, etc. [16,60]. These nanoparticles, with suitable bandgap and band-edge positions, are also used in water splitting reactions [5,61]. Semiconductor nanoparticles with their electronic and optic properties deployed by quantum mechanics are important materials used in electronic devices [44]. Due to the low thermal conductivity of nano silicon, it is used in photovoltaics, solar cells, electronics, and rechargeable batteries [22].

## 3. Methods for synthesis of nanomaterials

**Figure 2:**
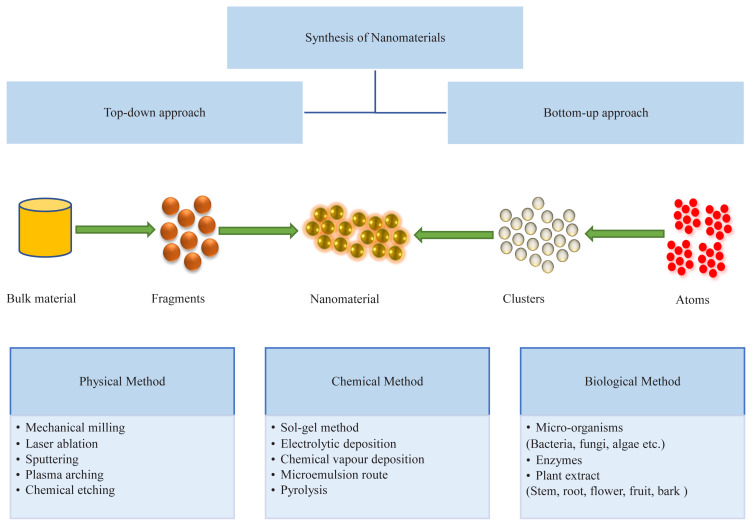
Methods for synthesis of nanoparticles.

Literature reports various methods that have been used to synthesize nanomaterials and can be categorized broadly into two types as illustrated in Figure 2 [14,28]. Using the top-down/destruction approach, nanoparticles are synthesized from bulkier molecules that are suitably disintegrated into smaller molecules and ultimately to the nanosized particles [62,63]. A variety of nanofabrication tools can be used to create nanostructures with the required shape and suitable characteristics from large molecules. These include mechanical milling, laser ablation, chemical etching, lithography, sputtering, etc. [7,41]. E.g., magnetic nanoparticles having a spherical shape with particles size varying from 80–130 nm were synthesized by coating with erythrocytes using a top-down approach [64]. Similarly, size-controlled drug nanoparticles of varying shapes have been obtained in nanosuspensions [62]. Researchers have also used milling method to obtain aqueous nanofluids containing silicon carbide nanoparticles [65].

In the bottom-up approach, simpler components (atoms or molecules) give rise to more complex nanoscale structures [66]. Therefore, this method is also known as the building-up/constructive approach [50]. Various nanofabrication methods in bottom-up synthesis include pyrolysis, chemical vapor deposition, sol-gel, spinning, biochemical and biological synthesis [7,67–69]. The approaches other than biological synthesis, require expensive instrumentation, enormous energy consumption, prolonged reaction time, and produce toxic by-products with the use of harmful and toxic ingredients leading to enhanced experimental cost and environmental pollution [13,34,70]. On the other hand, biological synthesis is preferable because of its more feasibility, less toxicity, low cost, and eco-friendly nature. Due to the use of biological compounds, this process is also termed biosynthesis or green synthesis [48,71]. These techniques are very useful for the generation of a multi-component system from simpler components without any elimination from the final system [18,46]. Recently, biosynthesis has been used for the synthesis of low-density lipoprotein nanoparticles for potential applications in drug delivery [53].

## 4. Green synthesis of nanoparticles

**Figure 3:**
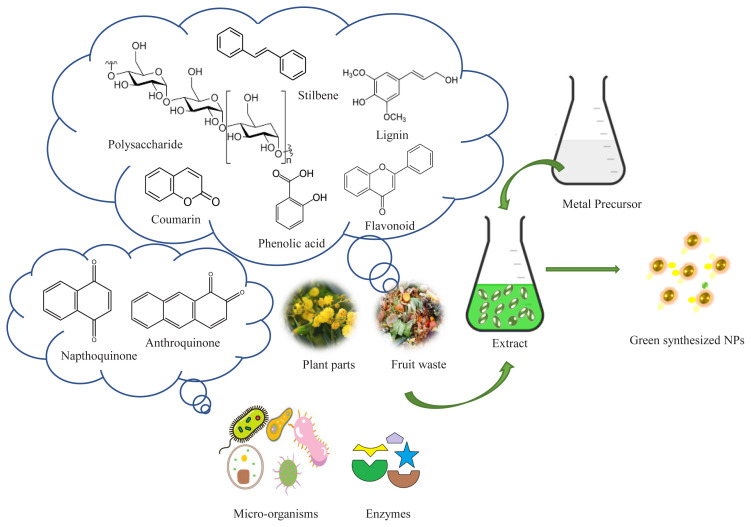
Mechanism for green synthesis of nanoparticles.

Inorganic nanoparticles have been produced using various biological substrates, including biomolecules (proteins, enzymes and DNA) [27,46,49,68,72,73], microorganisms (bacteria, fungi, and algae) [40,74] and plant extract (leaf, stem, root, flower, fruit and bark) [13,17,52,75,76]. The polyphenolic functionalities of the phytochemicals (polysaccharides, amino acids, proteins, enzymes, vitamins, organic acids, phenolics, terpenoids, tannins, saponins, and alkaloids, etc.) and biomolecules present in the biological extract, bind the metal ions and catalyze the reduction of metal ions to clusters of the zerovalent state that nucleate to form thermodynamically stable larger nanoparticles as illustrated in Figure 3 [77]. During the progression of this reaction, depending upon the pH value, reaction temperature, and nature of the reductant, nanoparticles of various sizes, and shapes are obtained [3,24,78]. The functionalities also stabilize the metal nanoparticles preventing their agglomeration and impart medicinal properties [61,79].

Researchers have used berry extract [80], leaf extract of *Mentha longifolia* [10], and red cabbage extract [81] for the synthesis of Au nanoparticles while leaf extract of *Cayratia edate* [6] and *Moringa oleifera* [61] have been explored for the synthesis of ZnO nanoparticles. Peel extract of *Artocarpus heterophyllus* was used to synthesize iron nanoparticles [1]. Silver nanoparticles have been synthesized by employing leaf extract of *Morus indica* L. V1 [82], fruit extract of *Terminalia chebula* [83], root extract of *Pelargonium endlicherianum* Fenzl. [84] as well as flower extract of *Cassia angustifolia* [85] and *Matricaria chamomilla* [86]. CuO nanoparticles have been synthesized using flower extract of *Matricaria chamomilla* [2] and peroxidase enzymes purified from *Ficus carica* leaves [15]. Vitamins and proteins present in quail egg yolk have been utilized for the synthesis of Pt [68] and Au [48] nanoparticles. DNA-based synthesis of hybrid nanoflowers has also been reported [87].

Studies reveal that endophytic fungi [88] and extracellular bacterial extract [89] have been exploited to synthesize Ag nanoparticles, while magnetic nanoparticles have been obtained using enzymes from *Acinetobacter calcoaceticus* [73] and *Pseudomonas putida* [3]. However, processes that utilize microbes are time-intensive due to the additional complicated stages involving microbial culture, isolation, and maintenance. On the other hand, plant extracts are more preferred because of easy availability, low cost, ease of handling, biodegradability, and involvement of simple techniques [7,42]. In addition, the synthesized nanoparticles can be easily collected and processed for characterization and potential applications [77].

## 5. Antimicrobial properties of nanoparticles

**Figure 4:**
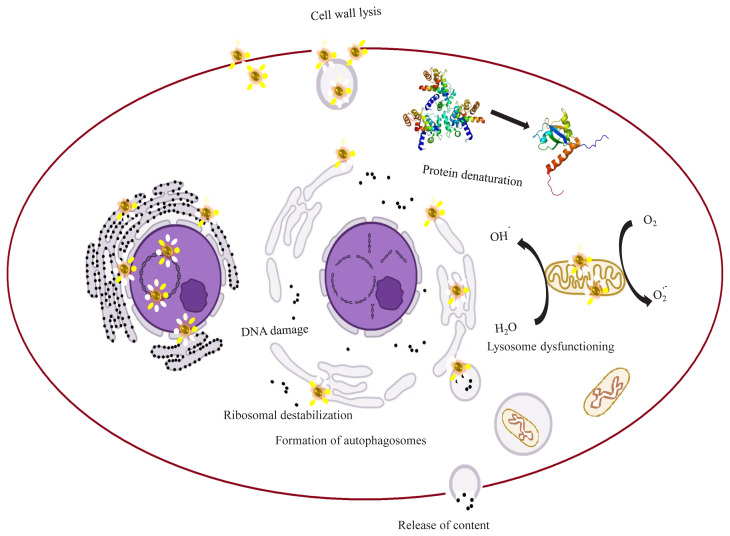
Mechanism for antibacterial action of nanoparticles.

As stated by the World Health Organization, antibiotic resistance has been among the key challenges to public health in the 21st century and currently available antibiotics are not enough to treat various bacterial infections. The solution is either the reduction of bacterial obstruction to antibiotics or using an alternative tool to fight against bacterial invasion [56]. In this context, nanoparticles have proven a promising tool with significant antimicrobial potential. Metal nanoparticles generate reactive oxygen species (ROS) that damage the cell membrane of bacteria and result in easier binding of nanoparticles to the bacterial surface resulting in their penetration inside the cell walls [33]. The nanoparticles cause denaturation of cellular proteins and enzymes resulting in loss of enzymatic action. The nanoparticles further interact with bacterial cell organelles such as DNA, ribosomes, mitochondria, and lysosomes leading to the disruption of normal cell functioning and result in metabolic imbalance proving lethal to the bacterial cell as illustrated in Figure 4 [76].

Antibacterial properties of nanoparticles vary as per their size, shape, and oxidation state. In general, d-block elements are potent antibacterial agents because of their high oxidation number and vital redox properties [35]. In addition, the ease of recoverability and recyclability potential of these fine particles extends their application in cosmetics, drug formulation, agri-food sector, waste-water treatment, etc. [13] Nanoparticles kill bacteria that are embedded in the extracellular polymeric substance (EPS) matrix of the biofilm [90]. Various metallic nanoparticles such as Ag, Au, and Pt show great antimicrobial activity. Silver nanoparticles exhibit significant antibacterial activity due to their small size and excellent hydrophilicity. Silver nanoparticles have been extensively explored as antibacterial as well as antifungal agents in cosmetics, drugs, and food-packaging films [55,91].

Nanoparticles are effective against various types of bacteria, algae, viruses, unicellular fungi, etc., and may be used in place of antibiotics. Additionally, the use of environmentally friendly metal nanoparticles synthesized through green processes has been shown to improve antibacterial activity against a variety of pathogens [89]. Biogenic nanoparticles exhibit enhanced applications for inhibiting pathogenic micro-organisms due to the synergistic effect of functionalities bound with the nanoparticles [16,58,74]. Green synthesized silver nanoparticles show enhanced antimicrobial activity against various pathogenic microorganisms, including bacteria and fungi. Biosynthesized silver nanoparticles using *Erythrina suberosa* leaf extract showed antibacterial activity against *Bacillus subtilis*, *Pseudomonas aeruginosa*, and *Staphylococcus aureus* [92] and that using identified antimicrobial molecules from *Pelargonium endlicherianum* Fenzl. root extract were found effective against *Escherichia coli*, *Pseudomonas aeruginosa*, and *Staphylococcus epidermidis* [84]. Significant antimicrobial activity of silver nanoparticles, synthesized from *Streptomyces violaceus* MM72, was observed against *Escherichia coli*, *Bacillus subtilis*, and *Pseudomonas aeruginosa* using the disc diffusion method [89]. The viability of *Xanthomonas perforans*, the causative agent for bacterial spot in tomatoes, was found to decrease significantly by DNA-assisted Ag-GO nanostructures [59,87].

Metal oxide nanoparticles of Fe, Cu, Zn, and Ti have been reported with significant catalytic and biomedical applications with anticancer, antimicrobial as well as antioxidant potential. The antimicrobial activity of iron nanoparticles, prepared by using *Eucalyptus robusta* extract, was evaluated by the agar diffusion method. These nanoparticles showed excellent antimicrobial activity against *Bacillus subtilis*, *Escherichia coli*, and *Staphylococcus aureus* [77]. Copper nanoparticles, synthesized using *Pseudomonas silesiensis* extract, showed good antimicrobial activity against *Escherichia coli*, *Bacillus cereus*, and *Staphylococcus aureus* [74]. Zinc oxide nanoparticles, synthesized using *Cassia auriculata* extract, showed potent antimicrobial action against *Escherichia coli*, *Staphylococcus aureus*, and *Streptococcus pneumonia* [76].

## 6. Applications of nanoparticles

**Figure 5:**
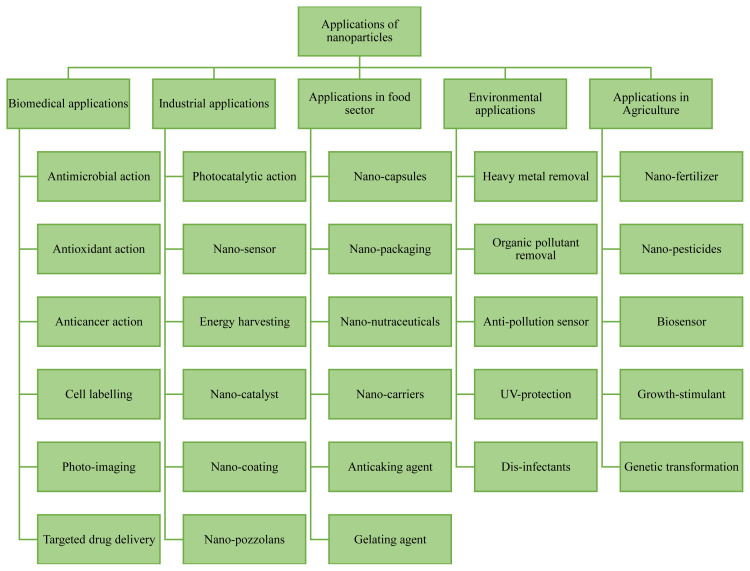
Applications of Nanoparticles in various sectors.

Nanoparticles offer explicit applications in different fields (Figure 5), including chemical, cosmetic, food processing, medical, construction, military, sports, textiles, electronics, automobile, and many more [52,53,55]. These applications are mainly accorded to their fine size (1 to 100 nm) and high specific ratio that leads to the improved manifestation of their chemical and physical properties as compared to conventional bulk materials [4,93]. Various single and multi-component nanoformulations are extensively explored in daily use employments like water and air purifiers, humidifiers, refrigerators, cosmetics, deodorants, and healthcare products due to their antibacterial properties [9,12,36]. Nanoparticle-based scaffolds are used in repairing damaged tissues through artificial stimulation of cell proliferation and implanting of organs [94]. Magnetic nanoparticles have potential applications in magnetic separation, enhanced oil and heavy oil recovery in addition to crude oil tracing with improved drilling and flow assurance [20,31]. Table lists the applications of some green synthesized nanoparticles, and the profound applications of nanoparticles in chemical, biomedical, and industrial sectors have been discussed ahead.

### 6.1. Catalytic applications

Nanoparticles have been used for the upgradation of electrochemical energy devices as well as for catalyzing different redox reactions [42]. Due to fine size, nanoparticles exhibit improved optical, electrical, and magnetic properties with significant applications in photodegradation and energy generation [76]. Nanoparticles are extensively used in photocatalytic water splitting, reduction of carbon dioxide, and piezoelectricity generation [44]. Carbon nanotubes and silver nanowires are used in light-emitting diodes to consume less energy [95]. Silver nanoparticles have been used for improving the redox potential of zeolites for better use in oxidation reactions with improved yield [96]. The catalytic activity of nanoparticles has identified their role as a more effective nanocatalyst [70]. Silver nanoparticles synthesized from the plant extract of *Ehretia laevis* exhibited significant catalytic activity towards the oxidation of alcohols into aldehydes [42]. Ag/Cu bimetallic nanoparticles synthesized using N_2_H_4_·H_2_O as reducing agent were effectively used as the catalyst for epoxidation of styrene [97]. The biosynthesized multicomponent nanoflowers have been found to mimic peroxidase activity [17,19] and have been used as Fenton’s reagent for oxidation of guaiacol [78,98]. Green synthesized iron nanoparticles were also found to act as Fenton’s reagent and were used for the degradation of Fuchsin basic dye [1]. Hybrid nanoflowers obtained by using Horseradish peroxidase were used to catalyze the polymerization of hydroquinone [23].

The catalytic activity of nanoparticles has also been explored in water treatment for the removal of harmful organic compounds. Biosynthesized silver nanoparticles were used for the effective reduction of methylene blue [83]. Nanocomposites of silver with TiO_2_ supported on cellulose acetate paper have been used for catalytic reduction of harmful organic pollutants including nitrophenols and dyes [32]. In another study, peroxidase-based copper nano-biocatalyst was found effective in decolorizing Victoria blue dye [24]. Researchers have also used red cabbage extract to synthesize gold nanoparticles with efficient potential for the reduction of nitrophenols [81]. Likewise, catecholamines directed hydrophilic gold nanoparticles were found to catalyze the reduction of nitrophenols more efficiently [79].

### 6.2. Water remediation

The rapid industrialization and enhanced agricultural practices, with massive population expansion, have contributed to the worldwide rise in environmental pollution. The release of domestic, agricultural, and industrial waste into nearby water resources causes an increase in the concentration of heavy metal ions and organic contaminants making the water unfit for use [5]. Further, the growth of pathogenic micro-organisms in wastewater is another issue of concern. Nanoparticles with antimicrobial, antioxidant, and photocatalytic potential show important applications in water remediation and are more effective than the conventional treatment methods [7]. Nanoparticles with enhanced surface activity not only remove the heavy metal ions by adsorption but also participate in catalytic reduction of organic matter and kill harmful micro-organisms.

Recently, magnetic nanoparticles have been used in wastewater treatment due to their many advantages in the disinfection and purification of wastewater. Magnetic nanoparticles with cost-effective preparation, high adsorption efficacy along with easy recoverability have been used for super magnetic separation of dyes and heavy metal ions from effluents [99]. Iron oxide nanoparticles have been used to remove Eu^3+^, La^3+^, Co^2+^, and other heavy metal ions as well as azo dyes [5]. Iron nanoparticles synthesized by using different leaf extracts have been explored for their potential to treat wastewater. It was observed that iron nanoparticles synthesized from *Azadiracta indica* extract showed significant efficacy to treat wastewater by removing phosphates and nitrates with an improvement of COD (chemical oxygen demand) [14]. Zero valent iron nanoparticles were used to efficiently treat distillery wastewater containing a high amount of inorganic and organic materials [99]. Most significantly, the low-cost, biodegradable, non-toxic, and bio-compatible chitosan nanoparticles with antimicrobial properties are used in multiple applications such as removal of dyes and heavy metal ions for wastewater treatment in addition to use in agri-food sector as anti-oxidant, enzyme inhibitor, and clarifying agent in food-packaging films [45]. Recently, silver nanoparticles have been used to disinfect potable water that has been considered safe for human consumption [100].

### 6.3. Industrial applications

Nanoparticles provide a diverse array of applications in various chemical-intensive industries. The materials produced with the use of nanoparticles are more durable, stronger, and lighter in weight. Hydrophobic SiO_2_ nanoparticles are used to obtain superhydrophobic surfaces with high abrasion resistance and anti-icing applications [43]. SiC nanoparticles are widely used for thermal insulation due to their high thermal stability, low thermal conductivity, and good corrosion resistance at elevated temperatures [65]. Nanoparticles are also used in mechanical industries in the form of alumina and titania nanocoatings that improve the toughness and wear resistance of materials [60]. Ultrahard and antireflective coatings of polysiloxanes have been prepared in presence of polymerizable nanoparticles using Boehmite nanoparticles as nanofillers [29]. Magnetic nanoparticles have been explored in reservoir sensing, drilling, and enhanced oil recovery with conformance control [31].

In the construction sector, various nanoparticles such as silica fume, fly ash, metakaolin, CNT, Al_2_O_3_, SiO_2_, TiO_2_, CuO, ZnO, and Fe_2_O_3_ have been used as supplementary cementitious materials in the form of an additive or partial substituent of cement in mortar and concrete structures [101]. These nanoparticles participate in pozzolanic and hydration reactions resulting in the production of extra calcium silicate hydrates (CSH) gel along with consumption of the detrimental calcium hydroxide in the cement matrix [41]. Consequently, the matrix microstructure gets improved resulting in enhanced mechanical strength and durability of the cementitious structures. The use of TiO_2_ and ZnO has been suggested to provide corrosion resistance and self-cleaning potential [102].

### 6.4. Biosensing applications

The conductive properties of various semiconductors and metallic nanoparticles have been applied in electroanalytical and biosensing [51]. Protein-synthesized gold nanoparticles were utilized for the detection of proteins in human urine and tears [25]. Similarly, multicomponent urease-based nanoparticles have been used for the detection of urea [46]. Gold and platinum nanoparticles have been used to modify surfaces of carbon electrodes with numerous applications in bioelectrochemistry, biosensors, and catalysis [103]. Silver nanoparticles coated platinum electrodes are used in surface-enhanced Raman spectral studies [11]. Noble metal nanoparticles are useful in chemical and biosensors due to their plasmon absorbance characteristics. Amino acid functionalized gold-liposomes nanostructures [69] exhibited efficient signal amplification potential for surface plasmon resonance biosensing while gallic acid-functionalized nanoflowers [67] exhibited significant potential for detection of m-cresols. Citrate-functionalized gold nanoparticles were used for label-free biosensing of macromolecules [22]. Acetylcholinesterase functionalized hybrid nano-flowers have been found highly efficient for the detection of pesticides. Enzyme–nanoparticle bio-conjugate were also used in bioassay activities i.e. wearing and microfluidic biosensors [27]. Metallic nanoparticles have exceptional quenching ability which makes them promising material for FRET (Forster resonance energy transfer) based biosensors that are used in sensing glutathione [95]. Light scattering technique based on plasmon resonance by gold nanoparticles has been used for studying the interaction between glycogen and biomacromolecules [104].

### 6.5. Diagnostic applications

The early detection of diseases can help to provide proper and timely treatment. Many non-invasive diagnostic techniques are available nowadays including optical imaging, MRI, and ultrasound imaging. Noble metallic nanoparticles have been utilized in in vivo imaging because of enhanced absorption of near-infrared light from biological tissue [28]. Multifunctional magnetic nanoparticles have been used to achieve desired stability, sensitivity, biocompatibility, and efficiency that are required for future medical diagnosis as well as for therapeutics [54]. Nanoparticles provide a direct analogy to the interaction of protein and carbohydrates by acting as an attractive multivalent receptor for various biomolecules [49]. It is important to detect genetic oligonucleotides sequence to diagnose genetic diseases which can be easily detected through the fabrication of gold nanoparticles [105]. Carbon nanotubes are useful in the detection of mutations in proteins and DNA [44]. Recently, metal nanoparticles and quantum dots have been used in the electrochemical, optical, and colorimetric detection of coronaviruses [12]. Recently, mannose-functionalized gold nanoparticles radiolabelled with technetium have been used as nanoprobes for the detection of lymph nodes [20].

Nanoparticles are also used in bioimaging, diagnostic sensing, and permeation enhancing that have been found useful for producing tumor site images. Many hydrophobic nanocrystals have been engineered to provide a molecular recognition function that can be utilized in the imaging of tumors [34]. DNA-passivated nanocrystals have been found to possess significant bioimaging applications with specific binding to proteins and nucleic acids [49]. Various noble-metal (Au and Ag) nanoparticles with biocompatibility and high electron density are used as contrast probes in computed tomography (CT) imaging [57]. Nanoparticles are also used in cellular, proteomic, and genomic labeling. Phase-transferred and thiol functionalized silver, gold, platinum, and palladium nanoparticles have been used in the formation of thin films and bio-labeling [63].

### 6.6. Drug delivery

Nanoparticles encapsulate the drug molecules and protect them from untimely degradation in addition to better binding with specific biomolecules to result in enhanced bioavailability at the specific target site [64]. Further, the effective clearance of the nanoparticles from the circulation system minimizes the toxic effects and results in the effective treatment of the patient with minimal side effects [9]. Micelles, dendrimers, liposomes, and polymeric nanoparticles are generally used for the designing of drug delivery systems [40]. Micelles with their amphiphilic nature improve the wettability and dissolution of drugs and have been extensively used to prepare stable nano-formulations that also provide ease of efficient drug release [62]. Dendrimers with an internal cavity and active functional groups at the surface act as promising theragnostic nanodevices for effective encapsulation and release of pH-sensitive drugs [54]. Liposomes with the lipid bilayer (mimicking phospholipid bilayer) and hydrophilic cavity not only enhance biocompatibility but also act as carriers for both hydrophilic as well as hydrophobic drugs [53]. Nowadays, lipid nanotechnology focuses on the designing and synthesis of lipid nanoparticles as a nanostructured mRNA carrier that can be used as a potential candidate for COVID-19 vaccines [12].

The intravenously injected drugs during chemotherapeutic pose a high risk with poor compliance and high toxic effects in patients. As such, direct delivery of the drug to the target tumorous cells is highly desirable. Nanoparticles have been extensively used for the targeted drug delivery in optimal dosage due to their fine size, better receptor binding, and increased stability [47]. Polymeric and biodegradable nanoparticles have been found useful in target drug delivery with biocompatibility and sustained release specifically in chemotherapy [64]. Naturally occurring pseudo-polymers named cellulose and chitin are also widely used as low-cost and biodegradable materials in antibacterial textiles, flexible displays, target drug delivery, and composites, etc. [47]. The ease of functionalization of bio-synthesized nanoparticles with improved binding with receptor molecules has enabled the targeted vaccine delivery to combat coronavirus [12]. The systematic functionalization with biocompatible molecules provides specific inhibitory potential along with minimizing the side effects. Paclitaxel encapsulated by albumin is valuable in chemotherapy for the treatment of breast and lung tumors under the trade name Abraxane [26]. Nano formulations such as Rapamune, Tricor, Triglide, and Avinza are excellent drug carriers and increase the bioavailability as well as the efficacy of water-insoluble drugs with high lipophilicity [62].

### 6.7. Cytotoxic potential

On the global scale, cancer is the leading cause of mortality with breast, lung, colon, and rectum cancer as the prominent cases. The limitations of existing chemotherapeutic drugs include poor hydrophilicity and cellular penetrability along with non-specific action causing severe side effects on the patient body. Further, in long duration, there is a development of resistance to these chemotherapeutic drugs resulting in decreased therapeutic response. In this context, biosynthesized nanoparticles with cytotoxic potential have been found effective in cancer treatment [2]. These nano-formulations with bioactive functionalities provide better inhibition of tumor proliferation with significant biocompatibility [28].

Cytotoxic activity of silver nanoparticles was evaluated for breast cancer cells using MTT (3-(4,5-dimethylthiazol-2-yl)-2,5-diphenyl tetrazolium bromide) assay [85]. Silver nanoparticles synthesized from *Pueraria tuberosa* aqueous extract showed the exceptional anti-cancer activity when evaluated using breast, brain, and multidrug resistant cancer cell lines [13]. Silver nanoparticles synthesized using leaf extract of *Carica papaya* showed anticancer effects when evaluated for HepG2, MCF-7, and A549 tumor cell lines [75]. Significant cytotoxic effect of zinc oxide nanoparticles, synthesized from the leaves of *Raphanus sativus var. Longipinnatus*, was observed for lung cancer cell line and were found to exhibit use as a potential chemopreventive agent for the cancer treatment [16]. Gold nanoparticles can be used to treat breast cancer of several types as gold nanoparticles show good anti-cancer effects. The anti-cancer property of gold nanoparticles was evaluated for breast infiltrating ductal cell carcinoma (Hs 319.T), adenocarcinoma (MCF7), and lobular carcinoma (UACC-3133) [10]. Gold nanoparticles, synthesized using leaf extract of *Anacardium occidentale*, showed selective cytotoxic effects towards MCF7 cancer cells [58]. Fabricated metal oxide nanoparticles of silicon [106] and iron [28] modified with amino functionalities have been used in gene targeting and in vitro tumor cell penetration, respectively. Conjugates of cisplatin with biosynthesized CoS nanoparticles were found to effectively reduce the proliferation of neuroblastoma cells [18].

## 7. Toxicity analysis

The toxicity concern of nanoparticles is an important aspect in consideration of the practical applications for technological advancements [37]. Nanoparticles enter our environment through various human activities and cause various toxicological effects on animal and plant cells [9]. There is uncertainty among researchers about the extent, effect, and threshold limit of toxicity for the safe use of nanoparticles. The toxic effects/health hazards may be due to exposure to nanoparticles during their production, storage, application, and improper disposal [107]. Reports suggest that nanoparticles upon interaction with body cells of an organism can have variable toxic effects, including inflammatory response and dysfunction of organs due to generation of oxidative stress with the ROS generation. Moreover, the toxic effect of nanoparticles is quite complex, and the extent of toxicity depends on their size, surface, physical, and chemical properties [33]. Generally, toxic effects of nanoparticles and size of nanoparticles have inverse relation, and ultrafine nanoparticles are more toxic than the particles of larger size with similar composition [38].

**Figure 6:**
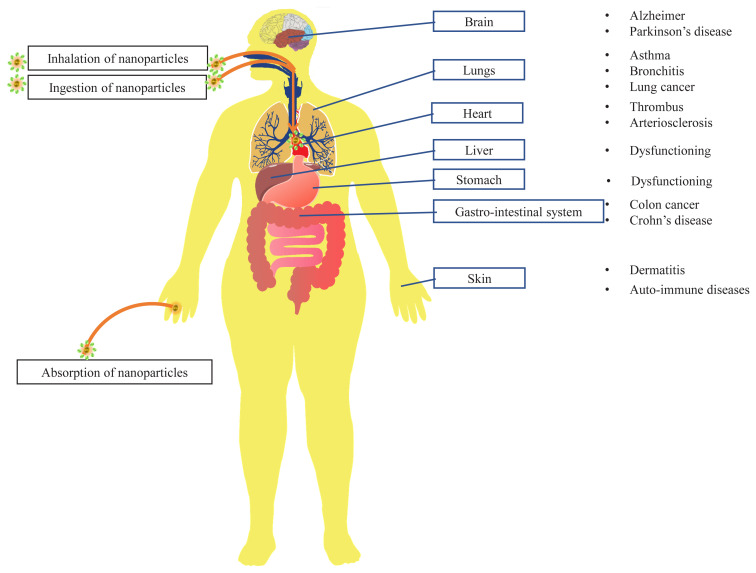
Toxic effects of nanoparticles on human body.

Since inhalation is the primary route for exposure to nanoparticles in human beings, their toxic effects are more prominent on the respiratory system, in addition to the effects on skin and other body organs due to dermal and oral exposure, as illustrated in Figure 6. Due to deposition in the respiratory tract, long-term exposure to nanoparticles such as CNT, asbestos and metal nanoparticles in various industries may lead to inflammation of the respiratory system, leading to tissue damage, tumors, and death [4]. The non-excreted as well as non-biodegradable nanoparticles migrate to the central nervous system through the olfactory nerve and accumulate in the brain, leading to chronic toxic effects including neurological diseases including Alzheimer’s and Parkinson’s disease [108].

The nanoparticles present in waste discharged into/near water resources can lead to their entry into aquatic flora and fauna, leading to a severe toxicological effect on these aquatic species, including increased lysis of cell walls, lysosomal activity, protein denaturation, enzyme deactivation, disturbance of electron transport system, ribosomal disassembly, chromosomal aberrations, and apoptosis [12, 74]. The presence of an excess amount of metal oxide nanoparticles has been found to exhibit similar cytotoxic and genotoxic effects. Aluminium oxide nanoparticles can damage cellular DNA, whereas zinc oxide nanoparticles have also been found to affect cell viability by interaction with DNA and electron transport systems [35]. Titanium oxide nanoparticles have been found to impose toxic effects on the liver, kidney, immune system, etc. [37] Various magnetic nanoparticles are used in bioengineering and cell imaging, but their long-term use can induce cytotoxicity. However, research has shown that chondrogenesis leads to degradation of these nanoparticles inside stem cells that get remagnetised, and only long-term exposure to excess dosage can induce irreparable damage [39]. Likewise, biosynthesized silver nanoparticles were found significantly effective against multidrug-resistant gut bacteria but were found safe for human cell lines [109].

## 8. Conclusion

The study explores the classification and synthetic techniques of nanomaterials with improved properties as compared to bulk materials. The antimicrobial properties of nanoparticles and applications of nanoparticles in various technological fields have been highlighted, with special emphasis on biosynthesized nanoparticles. During green synthesis by plant extracts, the nanoparticles get capped and stabilized by the functionalities of the phytochemicals present in the extract. As a result, the nanoparticles exhibit enhanced antimicrobial potential that is exploited in various biomedical applications. Nanoparticles are used for target-specific drug delivery and can serve as alternatives to traditional antibiotics, which have many drawbacks. The pioneering role of nanoparticles in chemotherapy and tumor diagnostics has provided a helping hand to scientists and practitioners. Nanoparticles can be tailored to get improved results in industrial applications and have provided new materials with improved mechanical, thermal, electrical, and durability properties. The photocatalytic action and redox potential have been well explored in synthesis, water splitting, and waste remediation. However, the improper handling and disposal of nanoparticles resulting in long-term exposure to excess content of nanoparticles can induce toxic effects on living organisms. Hence, suitable techniques must be developed to minimize the toxicity issues associated with nanoparticles, so that they can be conveniently engineered for beneficial purposes without any environmental or health concerns.

## Figures and Tables

**Table:** Applications of green-synthesized nanoparticles.

Extract		Nanoparticles				References
Type	Scientific Name	Type	Size	Shape	Applications	
Fruit peel	*Artocarpus heterophyllus*	Fe	33 nm	Spherical	Catalytic	[1]
Leaf	*Cayratia pedata*	ZnO	52.24 nm	Hexagonal	Enzyme immobilization	[6]
Leaf	*Mentha Longifolia*	Au	36.4 nm	Spherical	Anti-breast cancer	[10]
Tuber	*Pueraria tuberosa*	Ag	162.72 ± 5.02 nm	Spherical	Antioxidant and anticancer	[13]
Leaf	-	Fe	50–129 nm	Spherical	Treatment of domestic waste water	[14]
Leaf	*Raphanus sativus var. Longipinnatus*	ZnO	66.43 nm	Spherical	Anticancer	[16]
Leaf	*Ehretia laevis*	Ag	5–20 nm	Cubical, round	Catalytic	[42]
Leaf	*Anacardium occidentale*	Au	10–60 nm	Spherical	Antimicrobial and anticancer	[58]
Cell-free	*Pseudomonas silesiensis*	Cu	32 nm	Spherical	Antitumor and antimicrobial	[74]
Leaf	*Carica papaya*	Ag	12–28 nm.	Spherical	Antimicrobial and anticancer	[75]
Flower	*Cassia auriculata*	ZnO	41.25 nm	Flake	Antimicrobial and anticancer	[76]
Leaf	*Eucalyptus robusta*	Fe	8 nm	Spherical	Antioxidant and antimicrobial	[77]
Fruit	*Terminalia chebula*	Ag	25 nm	Spherical	Catalytic	[83]
Flower	*Cassia angustifolia*	Ag	10–80 nm	Spherical	Cytotoxic and antioxidant	[85]
Bacteria	*Streptomyces violaceus MM72*	Ag	30 nm	Spherical	Antimicrobial and antioxidant	[89]
Leaf	*Erythrina suberosa (Roxb.)*	Ag	73 nm	Spherical	Antimicrobial, antioxidant and cytotoxic activity	[92]
